# Efficacy of adjuvant-associated COVID-19 vaccines against SARS-CoV-2 variants of concern in randomized controlled trials: A systematic review and meta-analysis

**DOI:** 10.1097/MD.0000000000035201

**Published:** 2024-02-16

**Authors:** Meng-qun Cheng, Zhi-Ying Weng, Rong Li, Gao Song

**Affiliations:** a Department of Reproductive Medicine, The Pu’er People’s Hospital, Pu’er, China; b School of Pharmaceutical Science and Yunnan Key Laboratory of Pharmacology for Natural Products, Kunming Medical University, Kunming, China; c Department of Pharmacy, The Pu’er People’s Hospital, Pu’er, China.

**Keywords:** adjuvant vaccines, COVID-19 vaccines, efficacy, meta-analysis, randomized controlled trials (RCTs)

## Abstract

**Background:**

Adjuvants may enhance the efficacy of vaccines. however, the efficacy of adjuvant-associated COVID-19 vaccines (ACVs) remains unclear since the emergence of the COVID-19 pandemic. This study aimed to address this gap by conducting a systematic review and meta-analysis of the efficacy of ACVs against Severe Acute Respiratory Syndrome Coronavirus 2 CoV (SARS-CoV-2) variants of concern (VOC).

**Methods:**

A systematic search was conducted of randomized controlled trials (RCTs) evaluating the vaccine efficacy (VE) of ACVs against VOC (alpha, beta, gamma, delta, or Omicron), up to May 27, 2023. The DerSimonian-Laird random-effects model was used to assess VE with 95% confidence intervals (CI) through meta-analysis. Cochrane Risk of Bias tools were used to assess the risk of bias in RCTs.

**Results:**

Eight RCTs with 113,202 participants were included in the analysis, which incorporated 4 ACVs [Matrix-M (NVX-CoV2373), Alum (BBV152), CpG-1018/Alum (SCB-2019), and AS03 (CoVLP]). The pooled efficacy of full vaccination with ACVs against VOC was 88.0% (95% CI: 83.0–91.5). Full vaccination was effective against Alpha, Beta, Delta, and Gamma variants, with VE values of 93.66% (95% CI: 86.5–100.74), 64.70% (95% CI: 41.87–87.54), 75.95% (95% CI: 67.9–83.99), and 91.26% (95% CI: 84.35–98.17), respectively. Currently, there is a lack of RCT evidence regarding the efficacy of ACVs against the Omicron variant.

**Conclusion:**

In this meta-analysis, it should be that full vaccination with ACVs has high efficacy against Alpha or Gamma variants and moderate efficacy against Beta and Delta variants. Notably, with the exception of the aluminum-adjuvanted vaccine, the other ACVs had moderate to high efficacy against the SARS-CoV-2 variant. This raises concerns about the effectiveness of ACVs booster vaccinations against Omicron.

## 1. Introduction

Since the emergence of the novel severe acute respiratory syndrome coronavirus 2 (SARS-CoV-2) causing coronavirus disease 2019 (COVID-19) in December 2019, a total of 767 million confirmed cases and 6.93 million deaths have been reported globally as of May 31st, 2023.^[1]^ The development of COVID-19 vaccines has progressed rapidly, with 183 vaccines advancing to clinical trials and 199 vaccines in preclinical research as of March 2023.^[2]^ Among these,^[3–5]^ 33 contain different types of adjuvants, including 21 vaccines with protein subunits, 5 vaccines with virus-like particles, 5 vaccines are devised with inactivated whole viruses, 1 vaccine with non-replicating viral vectors, 1 vaccine with replicating viral vectors, and 1 combination vaccine with the Matrix-M adjuvant, containing both recombinant spike (rS) (SARS-CoV-2 rS) nanoparticles and influenza vaccine.

Like other viruses, SARS-CoV-2 has evolved over time, with notable variants of concern (VOC), including Alpha, Beta, Gamma, Delta, and Omicron.^[6]^ The Alpha variant was first identified in a UK case in 2020, followed by the beta variant in South Africa and the Gamma variant in Brazil, exacerbating the pandemic. In December 2020, the delta variant was discovered in India and neighboring countries, leading to a sharp increase in the number of cases and deaths. The Omicron variant of SARS-CoV-2 reportedly harbors over 30 mutations in its spike proteins, potentially leading to an apparent evasion of the immune response.^[7–10]^ Consequently, an in-depth evaluation of the efficacy of a vaccine specifically targeting the SARS-CoV-2 variant is necessitated.

There are systematic reviews on the efficacy of COVID-19 vaccines against SARS-CoV-2 variants of concern,^[11–15]^ but no studies have evaluated adjuvant-associated COVID-19 vaccines (ACVs) efficacy. Adjuvants play a key role in influenza, hepatitis, and malaria vaccine. In the post-pandemic era, adjuvants may be a breakthrough approach to address challenges such as high demand, decreased antibody titers, and reduced efficacy of some approved vaccines against variants.^[3]^ Therefore, to better understand the efficacy of ACVs, we systematically reviewed the evidence on the efficacy of ACVs against 5 SARS-CoV-2 VOC.

## 2. Methods

### 2.1. Data sources and searches

We conducted a systematic search of 4 databases, including PubMed, Embase, Cochrane Library, and Web of Science, using keywords such as “COVID-19,” “adjuvant,” “vaccine,” “variants,” and others, to retrieve relevant studies published up to May 27, 2023. EndNote X9.0 (Thomson ResearchSoft, Stanford) was used to manage records, conduct screening, and remove duplicates. This study adhered to the Preferred Reporting Items for Systematic reviews and Meta-Analyses (PRISMA) guidelines.^[16]^

### 2.2. Selection of studies

Our study included a systematic review and meta-analysis of randomized controlled trials (RCTs) that evaluated the efficacy of ACVs against 5 VOCs (alpha, beta, delta, gamma, and Omicron). Studies were excluded if they were: non-English language articles; observational studies, reviews, editorials, conference papers, case reports, or animal experiments; unconfirmed laboratory results; or studies that did not include samples with 2 dose vaccination schedule complete(full vaccination).

Two authors independently screened the titles and abstracts, and selected eligible studies based on these criteria. Any disagreements were resolved with the assistance of a third reviewer (the corresponding author).

### 2.3. Data extraction and quality assessment

The primary outcome of this study was the efficacy of the ACVs against VOC. The authors, CMQ, and LR independently extracted data using a predesigned Microsoft Excel 2016 spreadsheet, which included basic study information such as the first author, publication year, and journal name; study design features such as sample size, age, study location, number of participants, recruitment time, and study design; types of COVID-19 vaccines and adjuvants used; and efficacy results against VOCs. Vaccine efficacy (VE) was extracted using 95% confidence intervals (CI). The risk of bias for RCTs was assessed using the Cochrane Collaboration Network tool.^[17,18]^ Two investigators independently assessed the risk of study bias and a third investigator (SG) assisted in resolving any disagreements.

### 2.4. Statistical analysis

We conducted a DerSimonian-Laird random-effects meta-analysis to calculate pooled estimates and 95% CIs.^[19]^ The level of statistical heterogeneity among studies was assessed using the *I^2^* statistic, where *I^2^* values of 25%, 50%, and 75% represent low, moderate, and high heterogeneity, respectively.^[20]^ We performed subgroup analyses by study design blinding method, days after full vaccination (Day_F), and adjuvanted vaccine type. Subgroup comparisons were evaluated using a Q test, and statistical significance was set at *P* < .05. All analyses were conducted using the Stata 17.

## 3. Results

### 3.1. Literature search and study characteristics

**Figure 1.**
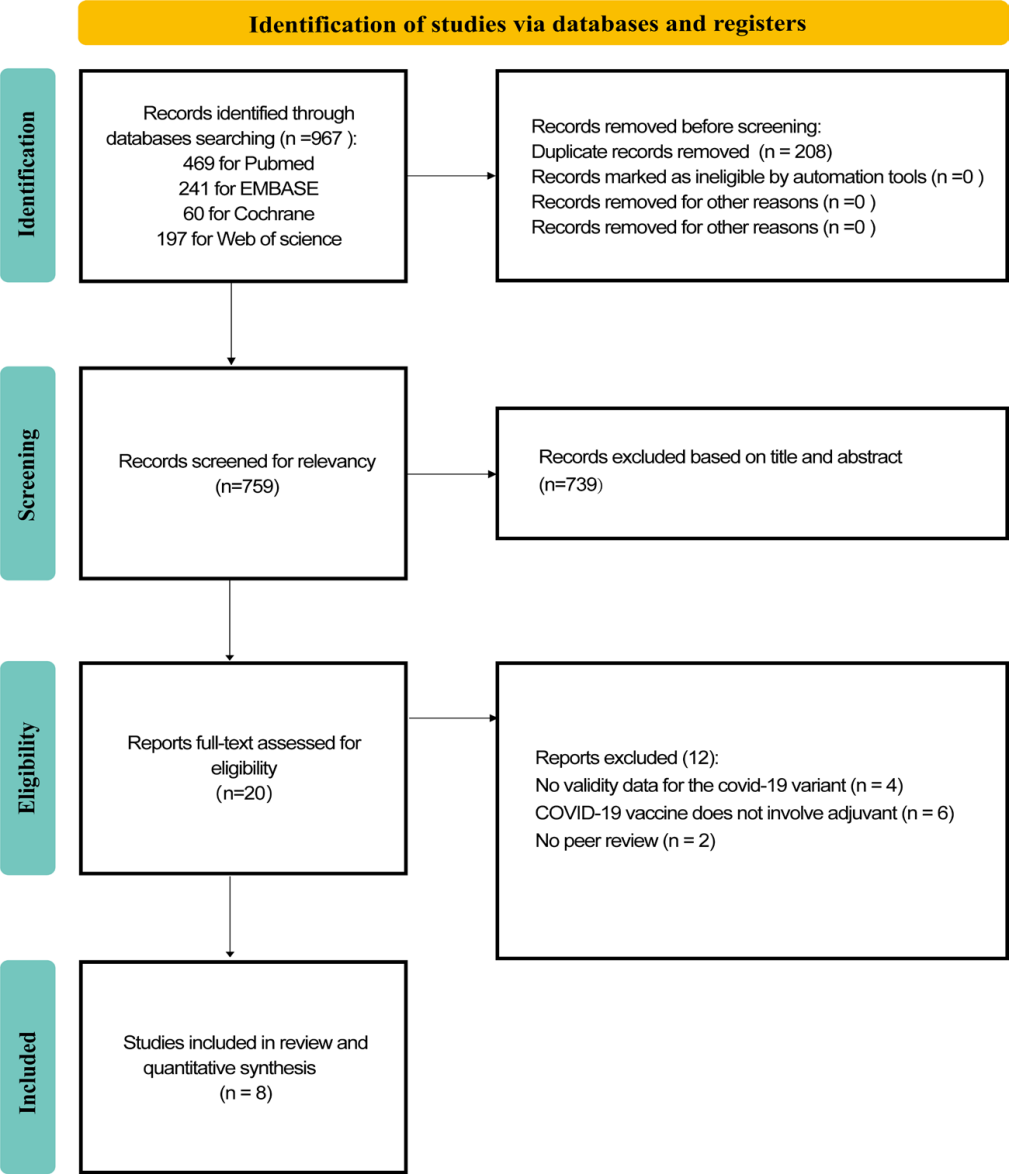
Flow chart of literature search and study selection.

A total of 967 records were retrieved from the 4 databases. After screening titles and abstracts, duplicates and articles unrelated to the topic were excluded, leaving 20 studies for full-text evaluation. Eventually, 8 studies that met the criteria were included in the systematic review and meta-analysis (Fig. 1).^[21–28]^ This study included 4 types of adjuvanted COVID-19 vaccines [Matrix-M(NVX-CoV2373),^[21,23,26,27]^ Alum(BBV152),^[24]^ CpG-1018/Alum(SCB-2019),^[22,28]^ AS03(CoVLP),^[25]^ and 5 VOCs (Alpha, Beta, Gamma, Delta, Omicron). Administration of the vaccine twice was considered full vaccination, and the study characteristics are summarized in Table 1. There were some concerns regarding the overall risk of bias assessment for all RCTs (see Table S1, http://links.lww.com/MD/L737, Supplemental Content, which illustrates the Risk of bias for included RCTs).

**Table 1:** Characterization of studies on the efficacy of the adjuvant COVID-19 vaccine.

Author	Journal	Study design (Phase; Blinding)	VOC*	Recruiting time	Age	Vaccine	Adjuvant type	No. of centers; country	N
Áñez (2023)^[21]^	JAMA Network Open	3; OB	Delta	2021.04.26–2021.06.05	12~17	NVX-CoV2373	Matrix-M	73; USA	1799
Bravo (2022)^[22]^	Lancet	2,3; DB	Delta(B.1.617.2); Gamma(P.1)	2021.03.24–2021.08.10	≥18	SCB-2019	CpG-1018/Alum	31; Belgium, Brazil, Colombia, Philippines and South Africa	11741
Dunkle (2021)^[23]^	N Engl J Med	3; OB	Alpha (B.1.1.7)	2020.12.27–2021.02.28	≥18	NVX-CoV2373	Matrix-M	119; USA & Mexico	25452
Hager (2022)^[25]^	N Engl J Med	3; OB	Alpha; Gamma; Delta;Omicron	2021.03.15–2021.09.02	≥18	CoVLP	AS03	85; Argentina, Brazil, Canada, Mexico, UK and USA	24141
Heath (2021)^[26]^	N Engl J Med	3; OB	Alpha(B.1.1.7)	2020.09.28–2020.11.28	18~84	NVX-CoV2373	Matrix-M	33; UK	14039
Smolenov (2022)^[28]^	Lancet Infect Dis	2,3; DB	Alpha(B.1.1.7); Beta(B.1.351, B.135.2,B.1.351.3); Delta(B.1.617.2); Gamma(P.1; P.1.1;P.1.2)	2023.03.24–2023.08.10	≥18	SCB-2019	CpG-1018/Alum	31; Belgium, Brazil, Colombia, Philippines and South Africa	14670
Shinde (2021)^[27]^	N Engl J Med	2a-b; OB	Beta(B.1.351)	2020.08.17–2020.09.25	18~64	NVX-CoV2373	Matrix-M	16; South Africa	4387
Ella (2021)^[24]^	Lancet	3; DB	Delta(B.1.617.2); Alpha(B.1.1.7)	2020.11.16–2021.1.7	≥18	BBV152	Alum	25; Indian	16973

DB = double blind, N = number of participants, OB = observer-blinded, RCT = randomized controlled trial, UK = United Kingdom, USA = United States, VOC = variants of concern.

* VOC were identified by genomic sequencing (GS) or variant circulation dominance.

### 3.2. Vaccine efficacy of various ACVs against VOCs

**Figure 2.**
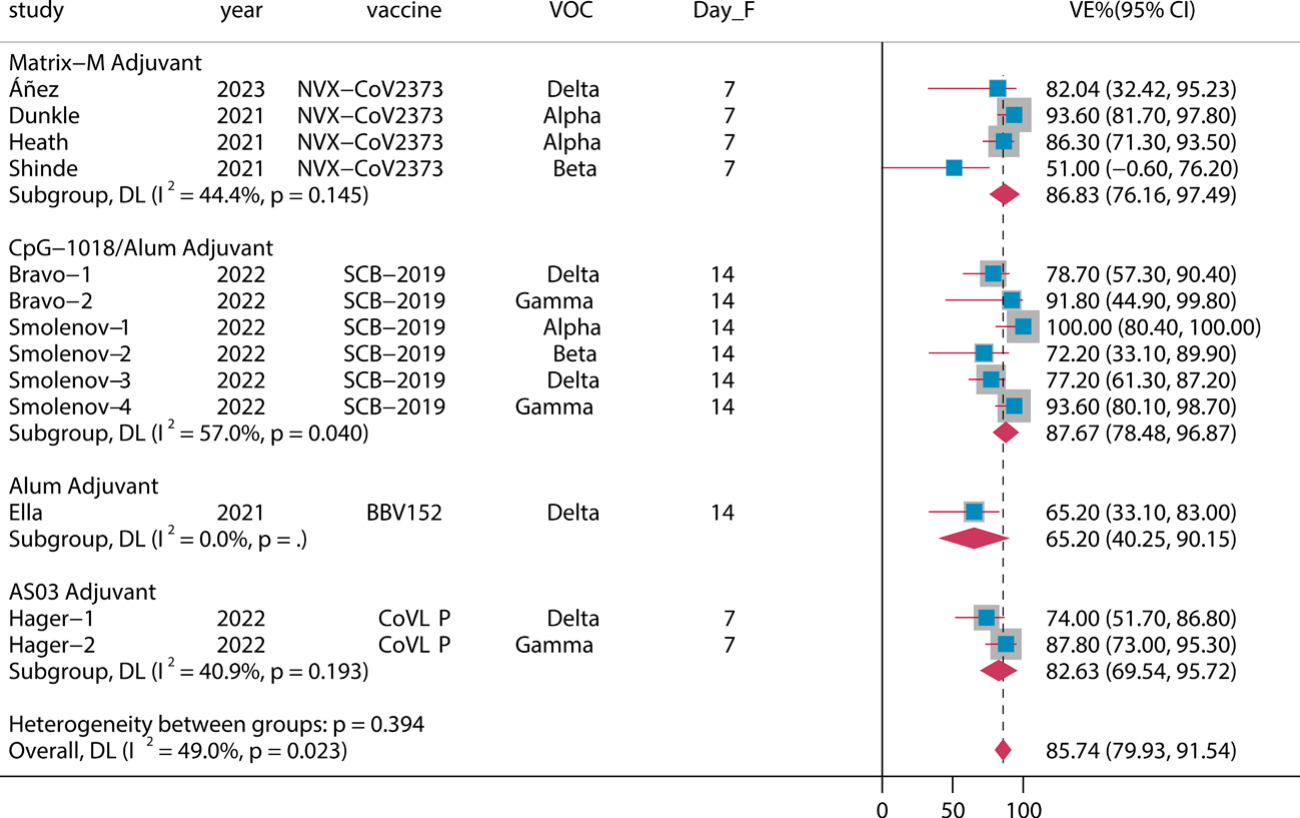
Vaccine efficacy of various ACVs against VOC. ACVs = adjuvant-associated COVID-19 vaccines, CI = confidence intervals, VE = vaccine efficacy, VOC = variants of concern, Day_F = days after the full vaccination.

Eight RCTs^[21–28]^ evaluated the efficacy of ACVs against VOC. Four types of ACVs were included in this analysis [matrix-M (NVX-CoV2373), alum (BBV152), CpG-1018/Alum(SCB-2019), and AS03(CoVLP)]. The summary VE of full vaccination against VOC was 85.74% (95% CI: 79.93–91.54) (Fig. 2). Subgroup analysis revealed that full vaccination was efficacious with AS03, alum, CpG-1018/alum, and Matrix-M against VOC, with VE of 82.63% (95% CI: 69.54–95.72), 65.20% (95% CI: 40.25–90.15), 87.67% (95% CI: 78.48–96.87), and 86.83% (95% CI: 76.16–97.49), respectively.

### 3.3. Vaccine efficacy of ACVs against Alpha variant

Of the 5 RCTs, 2 were not included in the meta-analysis due to missing VE data,^[24,25]^ resulting in a total of 3 RCTs^[23,26,28]^ assessing the efficacy of ACVs in preventing alpha variant infection. Two ACVs [NVX-CoV2373 (Matrix-M) and SCB-2019 (CpG-1018/Alum)] were included in the analysis. Subgroup results were included in one “double-blind” study and 2 “7 Day_F” studies (see Table S2, http://links.lww.com/MD/L738, Supplemental Content, which illustrates the VE of ACVs against Alpha variant). The VE of full vaccination against the alpha variant was 93.7% (95% CI: 86.6–100.7) (Table 2.). Subgroup analysis revealed a VE of 90.99% (95% CI: 84.13–97.85) for the subgroups of “Observer-blinded,” “7 d Day_F,” and “Matrix-M (NVX-CoV2373)” across the 2 RCTs. Additionally, in one RCT, a VE of 100.00% (95% CI: 80.40–100.00) was found among the subgroups of “Double Blind,” “14d,” and “CpG-1018/Alum(SCB-2019).” There appeared to be no significant difference in VE levels between the 2 ACVs in preventing alpha-variant infection (*P*_interaction_ = 0.324). The results of subgroup analyses are presented in Table 2.

**Table 2:** Meta-analysis and subgroup analysis of VE of ACVs against VOC.

Covariates	Subgroup	Study groups	I^2^	VE% (95% CI)	*P*(ES = 1)	*P*_interaction_
**Alpha**						
ALL		3	39.3%	93.66 (86.58–100.74)	<0.001	
Blinding	Observer-blinded	2	8.2%	90.99 (84.13–97.85)	0.023	0.324
	Double Blind	1	—	100.00 (80.40–100.00)	<0.001	
Day_F	7d	2	8.2%	90.99 (84.13–97.85)	0.023	0.324
	14d	1	—	100.00 (80.40–100.00)	<0.001	
Adjuvant vaccine type	Matrix-M (NVX-CoV2373)	2	8.2%	90.99 (84.13–97.85)	0.023	0.324
	CpG-1018/Alum (SCB-2019)	1	—	100.00 (80.40–100.00)	<0.001	
**Beta**						
ALL		2	0%	64.70 (41.87–87.54)	<0.001	
Blinding	Observer-blinded	1	—	51.00 (−0.60 to 76.20)	<0.001	0.384
	Double Blind	1	—	72.20 (33.10–89.90)	<0.001	
Day_F	7d	1	—	51.00 (−0.60 to 76.20)	<0.001	0.384
	14d	1	—	72.20 (33.10–89.90)	<0.001	
Adjuvant vaccine type	Matrix-M (NVX-CoV2373)	1	—	51.00 (−0.60 to 76.20)	<0.001	0.384
	CpG-1018/Alum (SCB-2019)	1	—	72.20 (33.10–89.90)	<0.001	
**Delta**						
ALL		5	0%	75.95 (67.91–83.99)	<0.001	
Blinding	Observer-blinded	2	0%	75.90 (60.58–91.22)	<0.001	0.890
	Double Blind	3	0%	75.97 (66.53–85.41)	<0.001	
Day_F	7d	2	0%	75.90 (60.58–91.22)	<0.001	0.890
	14d	3	0%	75.97 (66.53–85.41)	<0.001	
Adjuvant vaccine type	AS03 (CoVLP)	1	—	74.00 (51.70–86.80)	<0.001	0.710
	Alum (BBV152)	1	—	65.20 (33.10–83.00)	<0.001	
	Matrix-M (NVX-CoV2373)	1	—	82.00 (32.40–95.20)	<0.001	
	CpG-1018/Alum (SCB-2019)	2	0%	77.77 (67.57–87.97)	<0.001	
**Gamma**						
ALL		3	0%	91.26 (84.35–98.17)	<0.001	
Blinding	Observer-blinded	1	—	87.80 (76.65–98.95)	<0.001	0.402
	Double Blind	2	0%	93.41 (84.61–102.22)	<0.001	
Day_F	7d	1	—	87.80 (76.65–98.95)	<0.001	0.402
	14d	2	0%	93.41 (84.61–102.22)	<0.001	
Adjuvant vaccine type	AS03 (CoVLP)	1	—	87.80 (76.65–98.95)	<0.001	0.402
	CpG-1018/Alum (SCB-2019)	2	0%	93.41 (84.61–102.22)	<0.001	

ACVs = adjuvant-associated COVID-19 vaccines, CI = confidence intervals, Day_F = days after the full vaccination, VE = vaccine efficacy, VOC = variants of concern.

### 3.4. Vaccine efficacy of ACVs against beta variant

Two RCTs^[27,28]^ evaluated the efficacy of ACVs against beta-variants. Two ACVs were included in this analysis [Matrix-M (NVX-CoV2373) and CpG-1018/Alum (SCB-2019)]. Among them, the studies used 1 double-blind and 1 Observer-blinded, and 1 each of “7 d Day_F” or “14 d Day_F” (see Table S3, http://links.lww.com/MD/L739, Supplemental Content, which illustrates the VE of ACVs against Beta variant). The VE of full vaccination against the beta variant was 64.70% (95% CI: 41.87–87.54) (Table 2). Subgroup analysis revealed a vaccine efficacy (VE) of 72.20% (95% CI: 33.10–89.90) for the subgroups “Double Blind,” “14 Day_F,” and “CpG-1018/Alum(SCB-2019)” subgroups in 1 RCTs. There appeared to be no significant difference in VE levels between the 2 ACVs in preventing Beta variant infection (*P*_interaction_ = 0.384) (Table 2).

### 3.5. Vaccine efficacy of ACVs against delta variant

Five RCTs^[21,22,24,25,28]^ evaluated the efficacy of ACVs against Delta variants. Four ACVs were included in this analysis [Matrix-M (NVX-CoV2373), Alum (BBV152), CpG-1018/Alum (SCB-2019), and AS03 (CoVLP)]. Three studies were double-blind, and 2 were “7 Day_F” (see Table S4, http://links.lww.com/MD/L740, Supplemental Content, which illustrates the VE of ACVs against Delta variant). The summary VE of full vaccination against *Delta* variant was 75.95% (95% CI: 67.91–83.99) (Table 2.). Subgroup analysis revealed a VE of 75.90% (95% CI: 60.58–91.22) for the subgroups of “Observer-blinded” and “7 Day_F” across 2 RCTs. Additionally, in 3 RCT, a VE of 75.97% (95% CI: 66.53–85.41) was found among the subgroups of “Double Blind” and “14 Day_F.” There appeared to be no significant difference in VE levels among the 4 ACVs in preventing Delta variant infection (*P*_interaction_ = 0.71) (Table 2).

### 3.6. Vaccine efficacy of ACVs against Gamma or Omicron variant

Three RCTs^[22,25,28]^ evaluated the efficacy of ACVs against Gamma variants. omicron lacked VE data and was therefore not included in the meta-analysis.^[25]^ Two ACVs [AS03 (CoVLP) and SCB-2019 (CpG-1018/Alum)] were included in the analysis. One “double-blind” study and 2 “14 Day_F” studies (see Table S5, http://links.lww.com/MD/L741, Supplemental Content, which illustrates the VE of ACVs against Gamma or Omicron variant) were included in the subgroup analysis. The VE of full vaccination against the gamma variant was 91.26% (95% CI: 84.35–98.17) (Table 2). Subgroup analysis revealed a VE of 93.41% (95% CI: 84.61–102.22) for the subgroups of “Double Blind,” “14 Day_F,” and CpG-1018/Alum(SCB-2019) across 2 RCTs. Additionally, in one RCT, a VE of 87.80% (95% CI: 76.65–98.95) was found among the subgroups of “Observer-blinded,” “7 Day_F,” and “AS03 (CoVLP) “. There was no significant difference in VE levels between the 2 ACVs in preventing gamma variant infection (*P*_interaction_ = 0.402) (Table 2).

## 4. Discussion

In this systematic review and meta-analysis based on RCTs, we identified and included 8 RCTs that evaluated 4 different ACVs [Matrix-M(NVX-CoV2373), Alum(BBV152), CpG-1018/Alum(SCB-2019), AS03(CoVLP)] and 5 VOC (Alpha, Beta, Gamma, Delta, Omicron). We found that full vaccination with ACVs showed high levels of VE in preventing VOC, full vaccination with ACVs was highly effective against Alpha or Gamma variant infection and moderate efficacy against Beta or Delta variant infection, and more RCT evidence is needed to evaluate the VE of ACVs against Omicron variant infection. To our knowledge, this study is the first systematic review and meta-analysis to evaluate the evidence of ACVs against 5 VOCs.

COVID-19 low morbidity strategies are highly beneficial to public health, society and the economy, and indeed, concerted action is needed globally for the development of new multivalent vaccines and new variant vaccines(e.g., adjuvants), and we emphasize the importance of effective vaccine development strategies.^[29–31]^ SARS-CoV-2 has undergone evolution with the emergence of its new variants, characterized by enhanced transmissibility and the ability to at least partially evade neutralizing antibodies. At the same time, serum antibody levels begin to decline within a few months of vaccination, ultimately increasing the risk of breakthrough infections.^[32–35]^ Since 2022, the Omicron variant has become the major prevalent SARS-CoV-2 worldwide.^[36]^ COVID-19 vaccines are effective in preventing severe disease against Omicron and its sub-lineages, but their efficacy in preventing symptomatic infection is diminished.^[37,38]^ One study showed that Omicron, due to multiple spike mutations, escapes over 85% of tested neutralizing antibodies, posing a serious threat to existing COVID-19 vaccines.^[9]^ Currently, there is no RCT evidence on the efficacy of ACVs against Omicron infection. Our study showed that different ACVs have good protective efficacy against the Alpha, Beta, Gamma, and Delta variants. More RCT trials are needed to evaluate the efficacy of ACVs against Omicron infections.

Matrix-M is an adjuvant made from saponins extracted from the Quillaja saponaria molina tree, as well as cholesterol and phospholipids, and has been used in influenza vaccines^[39]^ and malaria vaccines.^[40]^ The Novavax COVID-19 vaccine (NVX-CoV2373) is a recombinant protein subunit vaccine composed of a trimeric spike glycoprotein and the Matrix-M1 adjuvant. Studies^[21,23,26}^ have shown that the efficacy of the NVX-CoV2373 vaccine against symptomatic COVID-19 is 80% to 90% in individuals aged 12 years and older, with specific efficacy influenced by age. Our study found that NVX-CoV2373 was moderately and highly protective in preventing symptomatic infections with Alpha and Delta variants. Notably, NVX-CoV2373 had the highest protective efficacy against symptomatic infection with the Delta variant strain in people aged 12 to 17 years (VE%: 82.04, 95% CI: 32.42–95.2).^[21]^ Krueger et al^[41]^ employed the VAccination Passes in Susceptible-Infectious-Recovered-Susceptible model (VAP-SIRS) to predict the efficacy of non-adjuvanted vaccines against Alpha and Delta variants. For the Delta variant, they determined the efficacies of Vaxzevria and Comirnaty as 0.6 and 0.79 respectively, and for Alpha variant, the assessed efficacies were 0.79 (Vaxzevria) and 0.92 (Comirnaty). Notably, the efficacy values derived from our study on adjuvanted vaccines seem to surpass these predicted values. Currently, whereas NVX-CoV2373 only contains antigens from the original SARS-CoV-2 strain and is not provided in a multivalent form. Similar to the Delta variant, the Omicron variant posed a higher risk of breakthrough infection. Therefore, the development of a multivalent NVX-CoV2373 targeting the Omicron variant is an important research direction.

S-Trimer (SCB-2019) is a recombinant protein subunit vaccine containing 2 adjuvants, the Toll-like receptor agonist CpG-1018 and alum.^[42]^ One study showed that^[43]^ 2 doses of SCB-2019, 3 weeks apart, elicited a strong virus-neutralizing antibody response with titers exceeding baseline levels for at least 6 months. Fourteen days after the second dose, the overall efficacy of the SCB-2019 vaccine against COVID-19 of any severity was 67.2% (95.72% CI 54.3–76.8), and the protective efficacy of previous exposure to SARS-CoV-2 could be improved to 83.2% (95% CI: 78.0–87.3).^[22,28]^ Our study showed that SCB-2019 was moderately or more effective in preventing infection with VOC (Alpha, Beta, Delta, and Gamma variants), especially for Alpha or Gamma, with protection levels exceeding 90% or more. The study of adjuvant-conjugated recombinant protein subunit vaccines has promising prospects.

CoVLP and BBV152 are inactivated COVID-19 vaccines, with the former utilizing the AS03 adjuvant and the latter utilizing the aluminum hydroxide adjuvant. AS03 is a squalene-in-water emulsion adjuvant containing α-tocopherol (vitamin E) as an additional immunoenhancing component, and has been approved for use in influenza vaccines. Studies have shown that, compared to non-adjuvanted vaccines, AS03 enhances the intensity and breadth of antibody and CD4^+^ T cell responses, thus enhancing protection against influenza.^[44]^ Aluminum hydroxide is the most commonly used aluminum adjuvant and is a traditional adjuvant widely used in human vaccines. Our research indicates that CoVLP (AS03 adjuvant) has moderate or greater protective effects against symptomatic infection from the Delta and Gamma variants. BBV152 (alum adjuvant) offered some protection against symptomatic infection from the delta variant (VE%: 65.20%, 95% CI: 33.10–83.00). The WHO guidelines recommend a lower limit of at least 30% and vaccine efficacy of at least 50%. Compared to other ACVs, BBV152 (alum adjuvant) had weaker protective efficacy, which may be related to the weaker ability of aluminum adjuvants to induce cellular immunity.

This study had certain limitations. First, although all the studies included in our analysis were RCTs, the control groups were placebo groups, and there was no direct head-to-head comparison with non-adjuvanted vaccines, which limited the scope of our study. In addition, owing to insufficient reporting and lack of data in the included studies, we were unable to assess the impact of VE on clinical outcomes related to hospitalization or death associated with VOCs. Furthermore, evidence for ACVs against the Omicron variant is still lacking and further research is needed in the future.

## 5. Conclusions

In this meta-analysis, it showed that the ACVs vaccine had high efficacy against Alpha or Gamma variants and moderate efficacy against Beta and Delta variants. With the exception of the alum-adjuvanted vaccine, the other ACVs were moderately and above effective against the SARS-CoV-2 variant strain. In summary, full vaccination with ACVs is important for the prevention of infection with SARS-CoV-2 variants. It is noteworthy that vaccination with ACV s may provide greater benefit against the current Omicron epidemic strain, especially in booster vaccination. However, there is a lack of relevant studies and the effectiveness of adjuvanted vaccines against Omicron variants needs to be further explored in real-world studies.

## Author contributions

**Conceptualization:** Meng-qun Cheng, Gao Song.

**Data curation:** Meng-qun Cheng, Gao Song.

**Formal analysis:** Meng-qun Cheng, Rong Li, Gao Song.

**Funding acquisition:** Gao Song.

**Investigation:** Meng-qun Cheng, Gao Song.

**Methodology:** Meng-qun Cheng, Rong Li, Gao Song.

**Project administration:** Meng-qun Cheng, Gao Song.

**Resources:** Meng-qun Cheng, Rong Li, Gao Song.

**Software:** Meng-qun Cheng, Rong Li, Gao Song.

**Supervision:** Meng-qun Cheng, Rong Li, Gao Song.

**Validation:** Meng-qun Cheng, Zhi-Ying Weng, Gao Song.

**Visualization:** Meng-qun Cheng, Gao Song.

**Writing – original draft:** Meng-qun Cheng, Gao Song.

**Writing – review & editing:** Meng-qun Cheng, Zhi-Ying Weng, Gao Song.

## Supplementary Material










