# Supplementary material for: Efficacy of adjuvant-associated COVID-19 vaccines against SARS-CoV-2 variants of concern in randomized controlled trials: A systematic review and meta-analysis
Source: Medicine (Baltimore). 2024 Feb 16;103(7):e35201. doi: 10.1097/MD.0000000000035201 (PMC10869057; doi:10.1097/MD.0000000000035201)
Supplement: Supplementary file 1 [file medi-103-e35201-s001.pdf]

Table S1. Risk of bias for included RCTs

Study (First author)	Randomization process	Deviations From Intended interventions	Missing outcome data	Measurement of the outcome	Selection of the reported result	Overall bias
Áñez（2023）	Low	Some concerns	Some concerns	Low	Some concerns	Some concerns
Bravo（2022）	Low	Some concerns	Low	Low	Low	Some concerns
Dunkle（2021）	Low	Some concerns	Low	Low	Low	Some concerns
Hager（2022）	Low	Some concerns	Some concerns	Low	Some concerns	Some concerns
Heath（2021）	Low	Some concerns	Some concerns	Low	Some concerns	Some concerns
Smolenov（2022）	Low	Some concerns	Some concerns	Low	Some concerns	Some concerns
Shinde（2021）	Low	Low	Some concerns	Low	Some concerns	Some concerns
Ella（2021）	Low	Some concerns	Some concerns	Low	Low	Some concerns

Note: The risk of bias for each domain can be classified into three levels: "low risk of bias," "some concerns," and "high risk of bias. The risk of bias for each domain can be classified into three levels: "low risk of bias", "some concerns" and "high risk of bias". If the risk of bias evaluation results in all areas are "low risk", then the overall risk of bias is "low risk"; if the risk of bias evaluation results in some areas are "some risk If there are areas where the risk of bias is evaluated as "moderate risk" and there are no areas with "high risk", then the overall risk of bias is "moderate risk"; as long as there is an area where the risk of bias is evaluated as "high risk", then the overall risk of bias is "moderate risk". "The overall risk of bias is "high risk".
