# Supplementary material for: Efficacy of adjuvant-associated COVID-19 vaccines against SARS-CoV-2 variants of concern in randomized controlled trials: A systematic review and meta-analysis
Source: Medicine (Baltimore). 2024 Feb 16;103(7):e35201. doi: 10.1097/MD.0000000000035201 (PMC10869057; doi:10.1097/MD.0000000000035201)
Supplement: Supplementary file 2 [file medi-103-e35201-s002.pdf]

Table S2. VE of ACVs against Alpha variant

Author	Country	Age	Vaccine	Adjuvant type	Control group	Blinding	Vaccination doses；interval	Day_F	Outcomes	VOC	n1/N1	n2/N2	VE%(95% CI)
Dunkle（2021）	USA & Mexico	≥18	NVX-CoV2373	Matrix-M	normal saline	OB	2; 21d	7d	Symptomatic	Alpha（B.1.1.7）	4/17308	27/8113	93.6(81.7~97.8)
Hager（2022）[a]	Argentina, Brazil, Canada, Mexico, UK and USA	≥18	CoVLP	AS03	Phosphate-buffered saline	OB	2; 21d	7d	Symptomatic	Alpha	0/12074	6/12061	100(38.2~NA)
Heath（2021）	UK	18~84	NVX-CoV2373	Matrix-M	normal saline	OB	2; 21d	7d	Symptomatic	Alpha(B.1.1.7)	8/7019	58/7009	86.3(71.3~93.5)
Smolenov（2022）	Belgium, Brazil, Colombia, Philippines and South	≥18	SCB-2019	CpG-1018/Alum	normal saline	DB	2; 21d	14d	All infections	Alpha(B.1.1.7)	0/7339	19/7312	100(80.4~100)

	Africa												
Ella（2021）[a]	Indian	≥18	BBV152	Alum	Algel formulation alone	DB	2; 21d	14d	Symptomatic	Alpha(B.1.1.7)	1/8470	3/8499	NA

[a]Missing data, not included in meta-analysis

Abbreviations: n1 Vaccinated people with SARS-CoV-2 infection; N1 Vaccinated people with no SARS-CoV-2 infection; n2 Unvaccinated people with SARS-CoV-2 infection; N2 Unvaccinated people with no SARS-CoV-2 infection; VE Vaccine efficacy; Day_F, days after the full vaccination; ACVs Adjuvant COVID-19 vaccines; OB Observer-blinded; DB Double-blinded; VOC variants of concern.
