# Supplementary material for: Efficacy of adjuvant-associated COVID-19 vaccines against SARS-CoV-2 variants of concern in randomized controlled trials: A systematic review and meta-analysis
Source: Medicine (Baltimore). 2024 Feb 16;103(7):e35201. doi: 10.1097/MD.0000000000035201 (PMC10869057; doi:10.1097/MD.0000000000035201)
Supplement: Supplementary file 3 [file medi-103-e35201-s003.pdf]

Table S3. VE of ACVs against Beta variant

Author	Country	Age	Vaccine	Adjuvant type	Control group	Blinding	Vaccination doses; interval	Day_F	Outcomes	VOC	n1/N1	n2/N2	VE%(95% CI)
Smolenov（2022）	Belgium, Brazil, Colombia, Philippines and South Africa	≥18	SCB-2019	CpG-1018/Alum	normal saline	DB	2; 21d	14d	All infections	Beta(B.1.351, B.135.2,B.1.351.3)	7/7332	23/7308	72.2(33.1~89.9)
Shinde（2021）	South Africa	18~64	NVX-CoV2373	Matrix-M	normal saline	OB	2; 21d	7d	Symptomatic	Beta(B.1.351)	11/2188	22/2166	51(-0.6~76.2)

Abbreviations: n1 Vaccinated people with SARS-CoV-2 infection; N1 Vaccinated people with no SARS-CoV-2 infection; n2 Unvaccinated people with SARS-CoV-2 infection; N2 Unvaccinated people with no SARS-CoV-2 infection; VE Vaccine efficacy; Day_F, days after the full vaccination; ACVs Adjuvant COVID-19 vaccines; OB Observer-blinded; DB Double-blinded; VOC variants of concern.
