# Supplementary material for: Efficacy of adjuvant-associated COVID-19 vaccines against SARS-CoV-2 variants of concern in randomized controlled trials: A systematic review and meta-analysis
Source: Medicine (Baltimore). 2024 Feb 16;103(7):e35201. doi: 10.1097/MD.0000000000035201 (PMC10869057; doi:10.1097/MD.0000000000035201)
Supplement: Supplementary file 4 [file medi-103-e35201-s004.pdf]

Table S4. VE of ACVs against Delta variant

Author	Country	Age	Vaccine	Adjuvant type	Control group	Blinding	Vaccination doses；interval	Day_F	Outcomes	VOC	n1/N1	n2/N2	VE%(95% CI)
Áñez（2023）	USA	12~17	NVX-CoV2373	Matrix-M	normal saline	OB	2; 21d	7d	Symptomatic	Delta	3/1202	8/586	82.04(32.42-95.23)
Bravo（2022）	Belgium, Brazil, Colombia, Philippines and South Africa	≥18	SCB-2019	CpG-1018/Alum	normal saline	DB	2; 21d	14d	All infections	Delta(B.1.617.2)	10/5925	46/5760	78.7(57·3~90·4)
Hager（2022）	Argentina, Brazil, Canada, Mexico, UK and USA	≥18	CoVLP	AS03	Phosphate-buffered saline	OB	2; 21d	7d	Symptomatic	Delta	12/12062	44/12023	74(51.7~86.8)

Smolenov（2022）	Belgium, Brazil, Colombia, Philippines and South Africa	≥18	SCB-2019	CpG-1018/ Alum	normal saline	DB	2; 21d	14d	All infections	Delta(B.1.617.2)	18/7321	72/7259	77.2(61.3~87.2)
Ella（2021）	Indian	≥18	BBV152	Alum	Algel formulation alone	DB	2; 21d	14d	Symptomatic	Delta(B.1.617.2)	13/8458	37/8465	65.2(33.1~83.0)

Abbreviations: n1 Vaccinated people with SARS-CoV-2 infection; N1 Vaccinated people with no SARS-CoV-2 infection; n2 Unvaccinated people with SARS-CoV-2 infection; N2 Unvaccinated people with no SARS-CoV-2 infection; VE Vaccine efficacy; Day_F, days after the full vaccination; ACVs Adjuvant COVID-19 vaccines; OB Observer-blinded; DB Double-blinded; VOC variants of concern.
