# Supplementary material for: Efficacy of adjuvant-associated COVID-19 vaccines against SARS-CoV-2 variants of concern in randomized controlled trials: A systematic review and meta-analysis
Source: Medicine (Baltimore). 2024 Feb 16;103(7):e35201. doi: 10.1097/MD.0000000000035201 (PMC10869057; doi:10.1097/MD.0000000000035201)
Supplement: Supplementary file 5 [file medi-103-e35201-s005.pdf]

Table S5. VE of ACVs against Gamma or Omicron variant

Author	Country	Age	Vaccine	Adjuvant type	Control group	blinding	Vaccination doses；interval	Day_F	Outcomes	VOC	n1/N1	n2/N2	VE%(95% CI)
Bravo（2022）	Belgium, Brazil, Colombia, Philippines and South Africa	≥18	SCB-2019	CpG-1018/Alum	normal saline	DB	2; 21d	14d	All infections	Gamma(P.1)	1/5934	12/5794	91.8(44·9~99·8)
Hager（2022）	Argentina, Brazil, Canada, Mexico, UK and USA	≥18	CoVLP	AS03	Phosphate-buffered saline	OB	2; 21d	7d	Symptomatic	Gamma	6/12068	47/12020	87.8(73.0~95.3)
Smolenov（2022）	Belgium, Brazil, Colombia, Philippines and	≥18	SCB-2019	CpG-1018/Alum	normal saline	DB	2; 21d	14d	All infections	Gamma(P.1; P.1.1;P.1.2)	3/7336	43/7288	93.6(80.1~98.7)

Hager （2022 ）[a]	South Africa Argentina, Brazil, Canada, Mexico, UK and USA	≥18	CoVLP	AS03	Phosphate-buffered saline	OB	2; 21d	7d	Symptomatic	Omicron	0/12074	0/12067	NA

[a]Missing data, not included in meta-analysis

Abbreviations: n1 Vaccinated people with SARS-CoV-2 infection; N1 Vaccinated people with no SARS-CoV-2 infection; n2 Unvaccinated people with SARS-CoV-2 infection; N2 Unvaccinated people with no SARS-CoV-2 infection; VE Vaccine efficacy; Day_F, days after the full vaccination; ACVs Adjuvant COVID-19 vaccines; OB Observer-blinded; DB Double-blinded; VOC variants of concern.
